# A commentary on ‘The effect of biofeedback pelvic floor training with ACTICORE1 on fecal incontinence: a prospective multicentric cohort pilot study’

**DOI:** 10.1097/JS9.0000000000001257

**Published:** 2024-03-05

**Authors:** Yanxia Hu, Huaxiang Deng, Guizhen Li, Jianxia Zhang, Mengying Zhang

**Affiliations:** Department of Gynaecology, Boao Yiling Care Center, Qionghai, Hainan, People’s Republic of China

*Dear Editor*,

The inadvertent loss of solid or liquid stool is known as faecal incontinence, and it affects 7% of individuals living in communities worldwide. It can significantly lower quality of life^1^. Paasch C *et al*.^2^ conducted a prospective nonrandomized multicentric clinical pilot investigation on patients with fecal incontinence who had received ACTICORE1 biofeedback training or daily Kegel exercise. Pelvic floor training with ACTICORE1 appears to offer sufficient pelvic floor training as a digital health application, and ACTICORE1 training compared to the Kegel exercise demonstrated noninferiority.

There were 40 people in all, four of whom dropped out, 19 of whom completed the ACTICORE1 training (ACTICORE-group) and 17 of whom underwent guideline-based physiotherapy (PHYSIO-group) between January 2020 and April 2021. Biometric parameter univariate analysis revealed no statistically significant variations. The study discovered that the ACTICORE-group members were, on average, younger (M=46.6 (SD=18.9) years compared to M=57.1 (SD=17.3) years, *P*=0.093). Regarding the assessment of endpoints, it was found that ACTICORE1 did not perform less poorly than the therapy standard, which is Kegel exercise. After 16 weeks, Wexner scoring indicated a statistically significant intraindividual improvement in fecal incontinence in both groups. With equivalency boundaries of five for both low and high, the TOST found that the ACTICORE1 training was not inferior (98% CI; results: 1.36, upper 6.75).

There are a lot of advantages and inspirations in this essay. One of the trial’s advantages was that it was a prospective, multicenter, controlled study with a suitable sample size, sufficient to identify any clinically significant differences between groups. Thus, the research offers reliable results to guide treatment. Participants were drawn from a variety of outpatient and community settings, and the two groups were highly comparable at the outset, enhancing the generalizability of our findings. Still, there were a lot of limitations. First, there was a potential of selection and detection bias since the nonblinded investigator and missing randomization failed to conceal group assignment from participants, therapists, or researchers^3^. Second, the lack of some clinical characteristics, such as a history of illness, led to bias. The primary risk factors for faecal incontinence are bowel disturbances, especially diarrhoea, anal sphincter trauma (from obstetrical injury or prior surgery), rectal urgency, and the burden of chronic illness^4^. Other risk factors include neurological disorders, inflammatory bowel disease, and pelvic floor anatomical disturbances. Ultimately, objective data can be obtained by considering anorectal manometry, which measures resting pressure and maximum squeeze increment pressure, rectal capacity measurements, which measure sensory threshold, urge sensation, and maximum tolerable volume, and fecal incontinence episodes recorded through a 14-day bowel diary^5^.

The purpose of this study is to compare the efficacy of physiotherapeutically guided PFMT with ACTICORE1 biofeedback training. There are significant implications for clinical practice from this issue. In addition to showing noninferiority when comparing ACTICORE1 training to Kegel exercise, this prospective nonrandomized multicentric clinical pilot study offers preliminary evidence that pelvic floor training using ACTICORE1 may enable adequate pelvic floor training as a digital health application. This creative approach provides enlightening guidance and inspiration for the continued development of this area of research.

## Ethical approval

This manuscript is a comment. Do not need ethical approval.

## Consent

This manuscript is a comment. Do not need patients consent.

## Author contribution

Y.H.: study concept or design, data collection, data analysis or interpretation, and writing the paper; H.D.: study concept or design, data collection, data analysis or interpretation, and writing the paper; G.L. and J.Z.: data analysis or interpretation; M.Z.: study concept or design, writing, and revise the paper.

Co-authors: Yanxia Hu and Huaxiang Deng equally contributed to the article.

## Conflicts of interest disclosure

This manuscript is a comment without conflicts of interest.

## Research registration unique identifying number (UIN)

This manuscript is a comment. Do not need UIN.

## Guarantor

Mengying Zhang.

## Data availability statement

This manuscript is a comment. Do not need data availability statement. But all the data during the current study are publicly available.

## Provenance and peer review

This manuscript is a comment without being invited.
